# Supplementary material for: Long-read-sequenced reference genomes of the seven major lineages of enterotoxigenic Escherichia coli (ETEC) circulating in modern time
Source: Sci Rep. 2021 Apr 29;11:9256. doi: 10.1038/s41598-021-88316-2 (PMC8085198; doi:10.1038/s41598-021-88316-2)
Supplement: Supplementary file 1 — Supplementary Information 1. [file 41598_2021_88316_MOESM1_ESM.pdf]

Additional file 1
Supplementary Figures and Tables
Figures S1-S16
Tables S1-S4

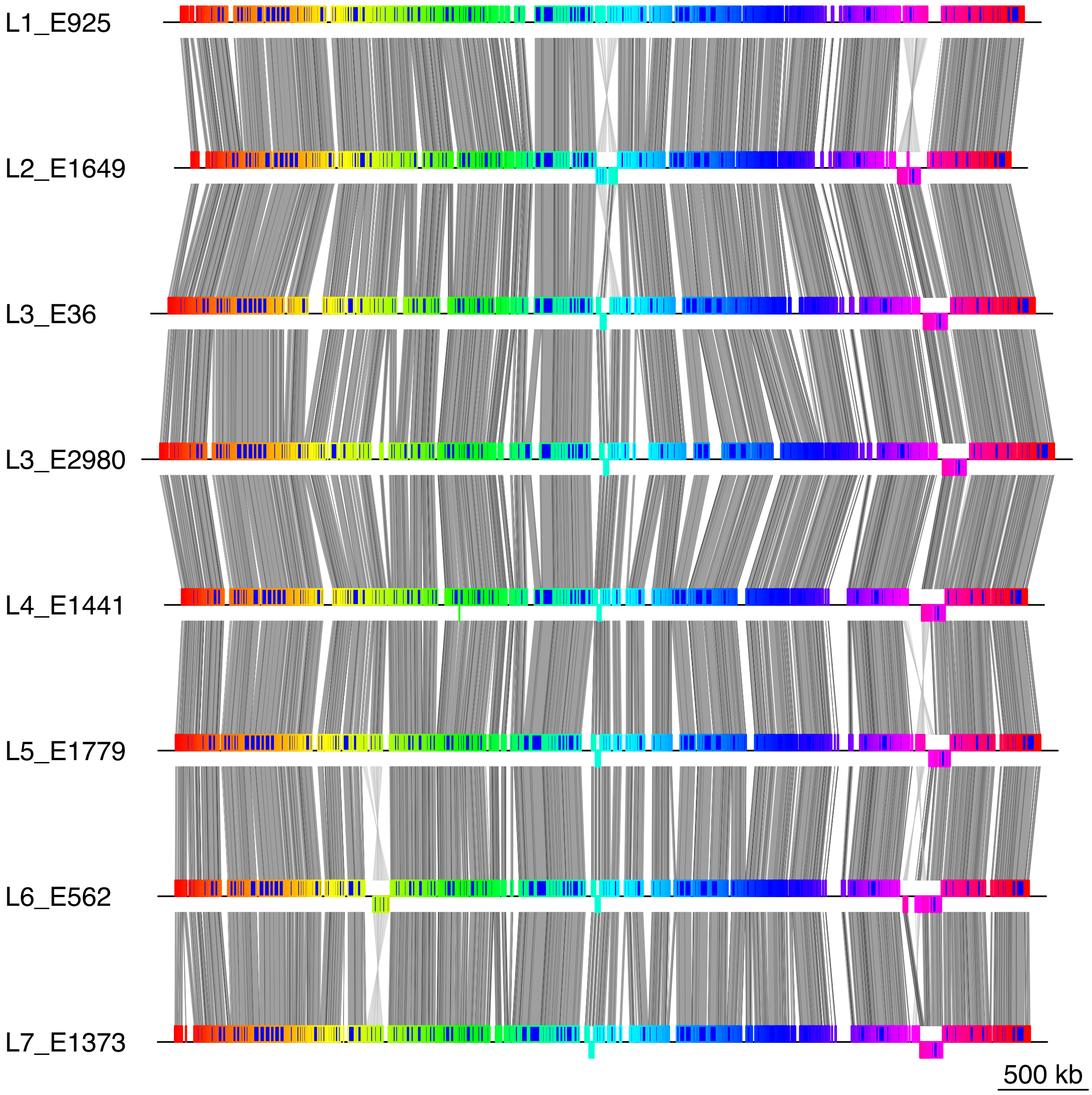

Figure S1: Comparison of the chromosomes of each ETEC reference genome using progressiveMAUVE. Circularized chromosomes extracted from the reference ETEC strains of lineage 1 through 7 (two reference strains belong to L3, one is CFA/I and the other CS7). The chromosomes are to a large extent colinear. Figure visualised using R (v4.0.2, 2020-06-22, URL: https://www.R-project.org/), specifically the R package genoplotR.

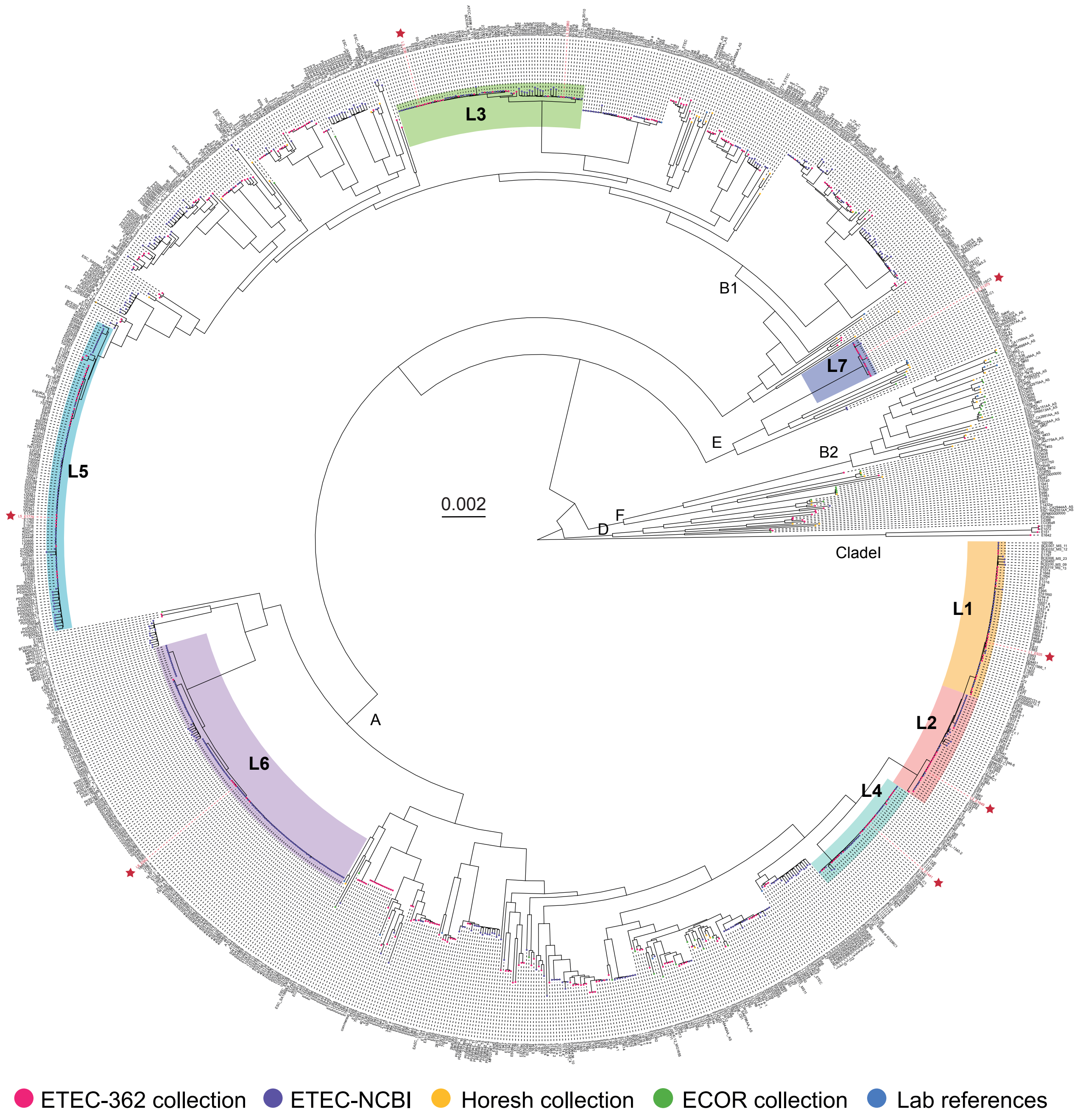

Figure S2: Phylogenetic analysis of ETEC genomes in context of *E. coli* genomes. Phylogenetic tree and network depicting the relationship of genomes from multiple ETEC studies (refs. 18, 20, 24, 26, 27, 47, 80-81) and representative *E. coli* genomes including *E. coli* commensals and pathotypes (refs. 78 and 79). Maximum likelihood phylogeny (1000 ultra fast parametric bootstraps, midpoint rooted). Strain IDs are located on the tips and the ETEC reference strains are colored in red and marked with a red star. The phylogroups are indicated in the tree and the major lineages presented in ref. 18 are highlighted. The scale bar indicates the number of substitutions per site. A tree with bootstrap values is provided in Figure S11. The tree was visualised with R (v4.0.2, 2020-06-22, URL: https://www.R-project.org/), specifically using the R packages GGTREE (v2.4.1, URL: https://github.com/YuLab-SMU/ggtree) and GGPLOT2 (v3.3.2, URL: https://ggplot2.tidyverse.org).

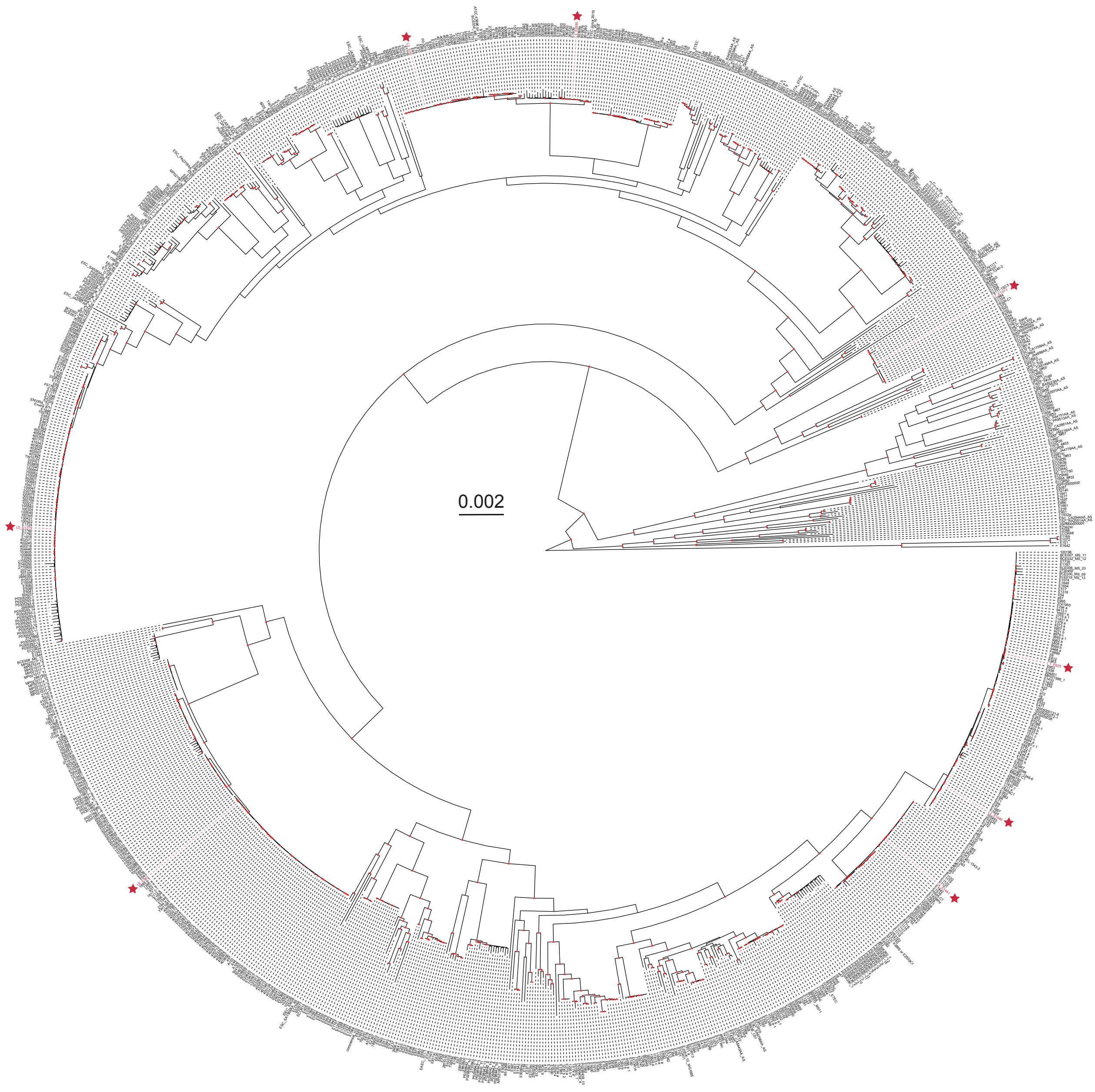

Figure S3: Bootstrap values of the phylogenetic tree of ETEC and *E. coli* isolates. Maximum likelihood phylogeny (1000 ultra fast parametric bootstraps, midpoint rooted). Nodes with a bootstrap values >95 are marked with a red triangle. Strain IDs are located on the tips and the ETEC reference strains are colored in red and marked with a red star. The scale bar indicates the number of substitutions per site. The tree was visualised with R (2020, URL:), specifically using the R packages GGTREE (v2.4.1, URL: https://github.com/YuLab-SMU/ggtree) and GGPLOT2 (v3.3.2, URL: https://ggplot2.tidyverse.org).

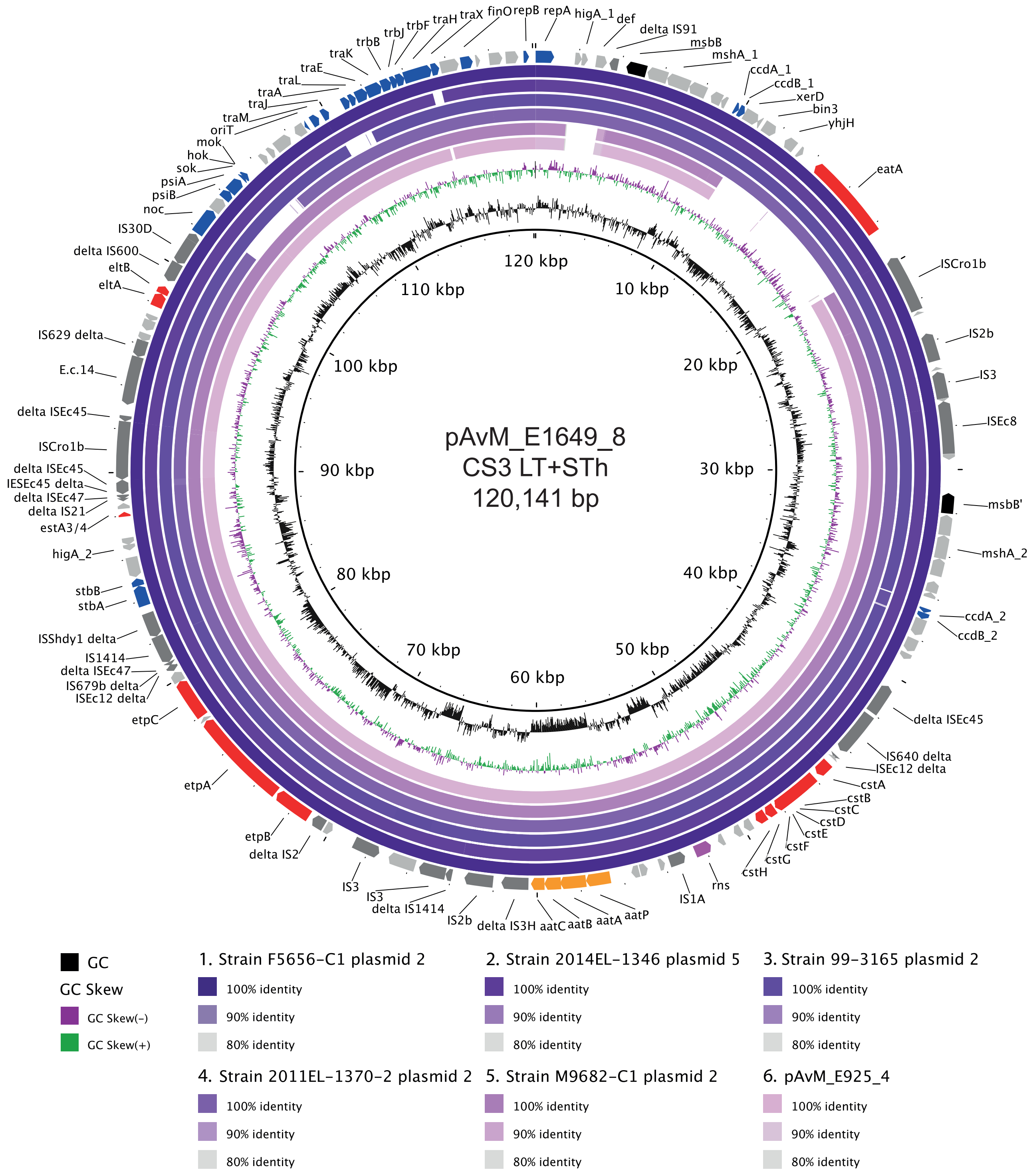

Figure S4: Comparison between the ETEC reference plasmids harbouring colonisation factors and other PacBio-sequenced ETEC plasmids using blastn. Plasmid pAvM_E1649_8 (CS3) as reference and pAvM_925_4 (CS3) compared to the following ETEC plasmids: F5656-C1 plasmid 2 (USA, CP024262.1), 2014-EL-1346-6 plasmid 5 (2014, USA; CP024237.1), 99-3165 plasmid 2 (USA; CP029980.1), 2011EL-1370-2 plasmid 2 (2011, USA; CP022914.1) and M9682-C1 plasmid 2 (1975, USA; CP024277.1). The figure was generated using BRIG (v0.95, URL: http://brig.sourceforge.net/).

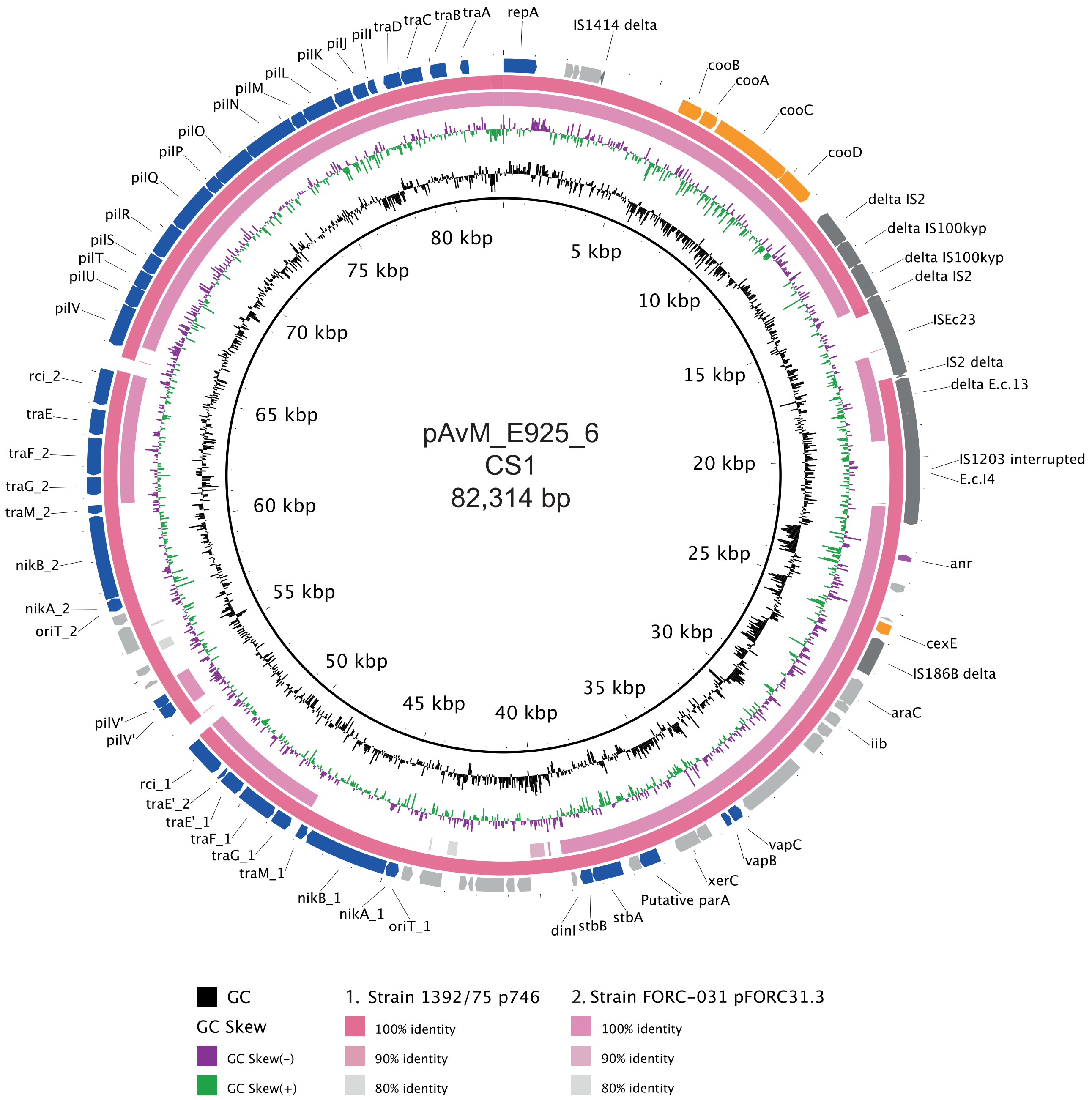

Figure S5: Comparison between the ETEC reference plasmids harbouring colonisation factors and other PacBio-sequenced ETEC plasmids using blastn. Plasmid pAvM_E925_6 (CS1) compared to ETEC plasmids 1392/75 p746 (1973; FN822748.1) and pFORC31.3 (2004, Korea; CP013193.1). The figure was generated using BRIG (v0.95, URL: http://brig.sourceforge.net/).

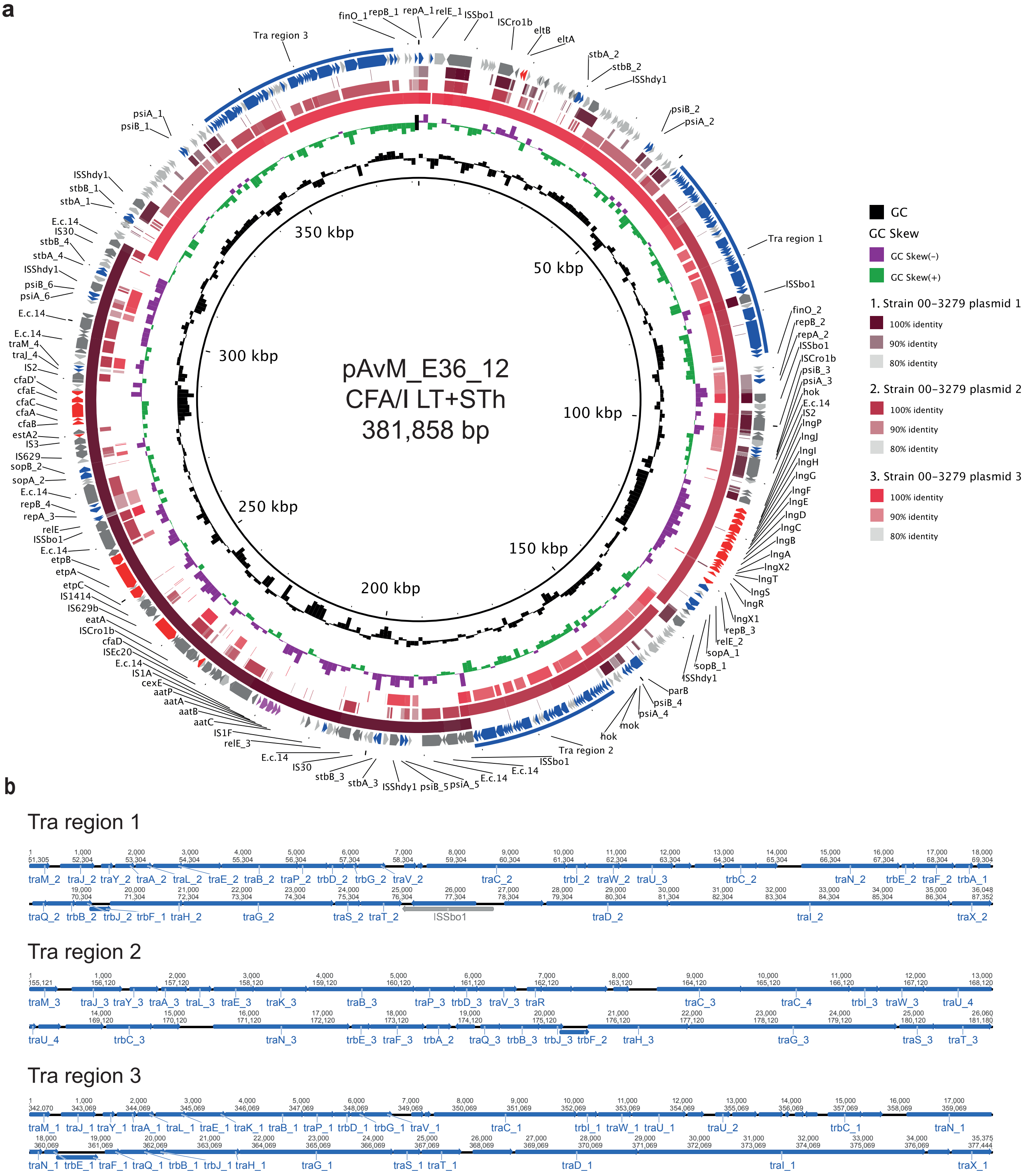

Figure S6: Comparison between the ETEC reference plasmids harbouring colonisation factors and other PacBio-sequenced ETEC plasmids using blastn. **a)** pAvM_E36_12 (CFA/I) compared to plasmids 1-3 (p1: CP024294.1; p2 CP024295.1; p3: CP024296.1) from ETEC strain 00-3279 (USA). **b)** Organisation of Tra regions 1, 2 and 3. Figure S6a was generated using BRIG (v0.95, URL: http://brig.sourceforge.net/) and Figure S6b was generated using Genious (v11.1.5, URL: http://www.geneious.com).

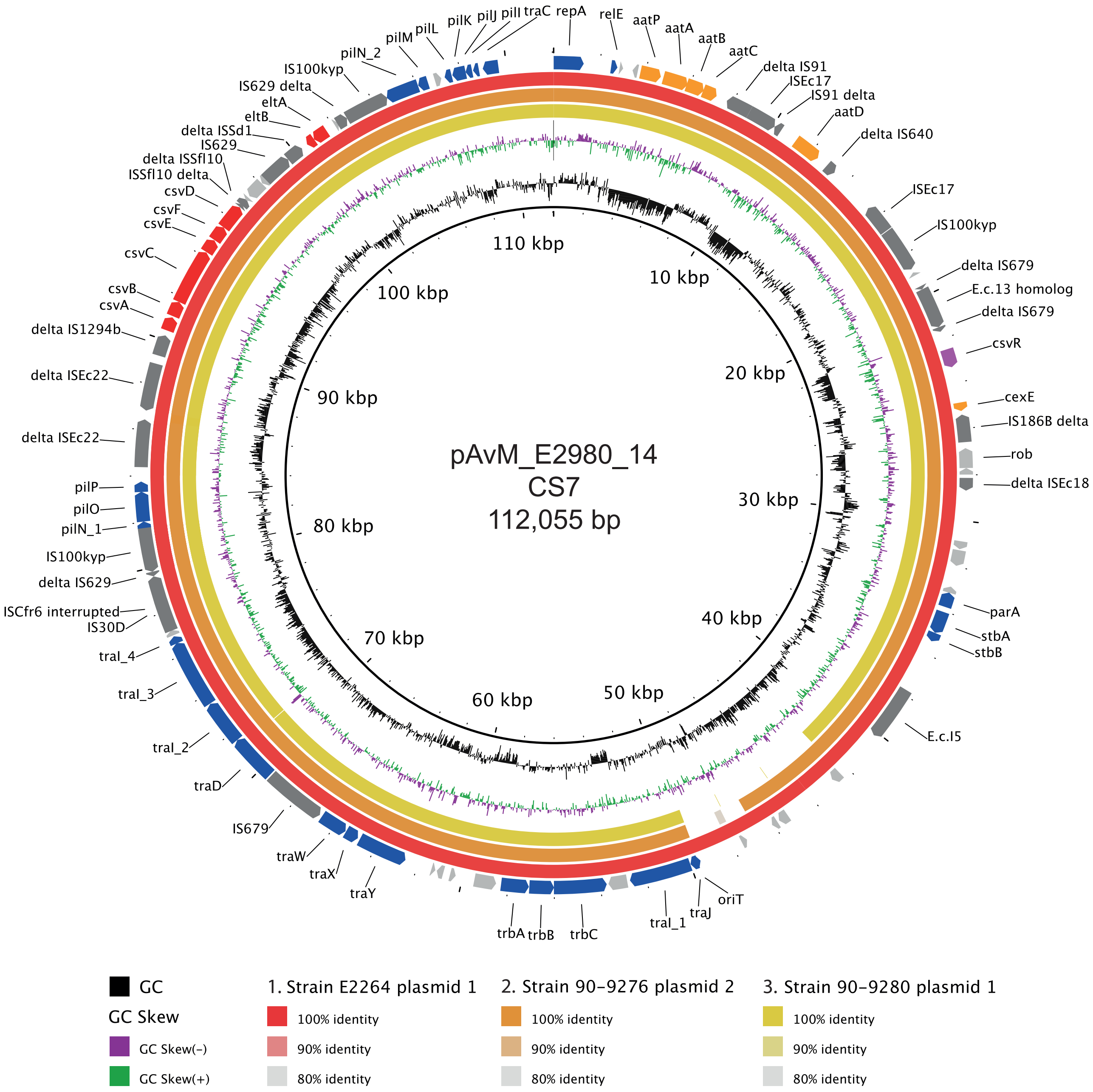

Figure S7: Comparison between the ETEC reference plasmids harbouring colonisation factors and other PacBio-sequenced ETEC plasmids using blastn. Plasmid pAvM_E2980_14 (CS7) compared to E2264 plasmid 1 (2006, Bangladesh; CP023350.1), 90-9276 plasmid 2 (1988, Bangladesh; CP024298.1) and 90-9280 plasmid 1 (1988, Bangladesh; CP024241.1). The figure was generated using BRIG (v0.95, URL: http://brig.sourceforge.net/).

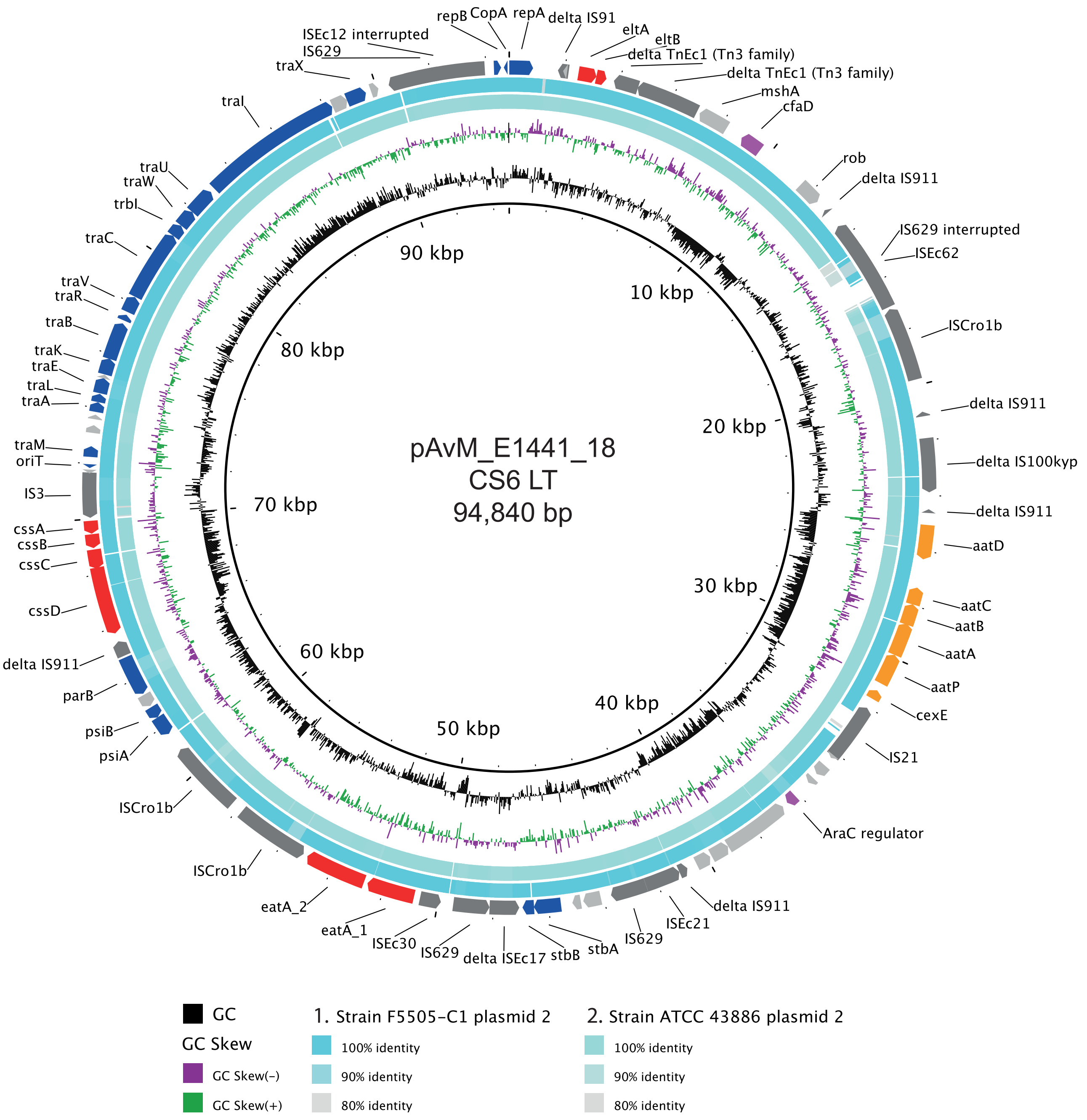

Figure S8: Comparison between the ETEC reference plasmids harbouring colonisation factors and other PacBio-sequenced ETEC plasmids using blastn. pAvM_E1441_18 (CS6) compared to F5505-C1 plasmid 2 (2013, Sweden; CP023259.1) and ATCC 43886 plasmid 2 (CP024255.1). The figure was generated using BRIG (v0.95, URL: http://brig.sourceforge.net/).

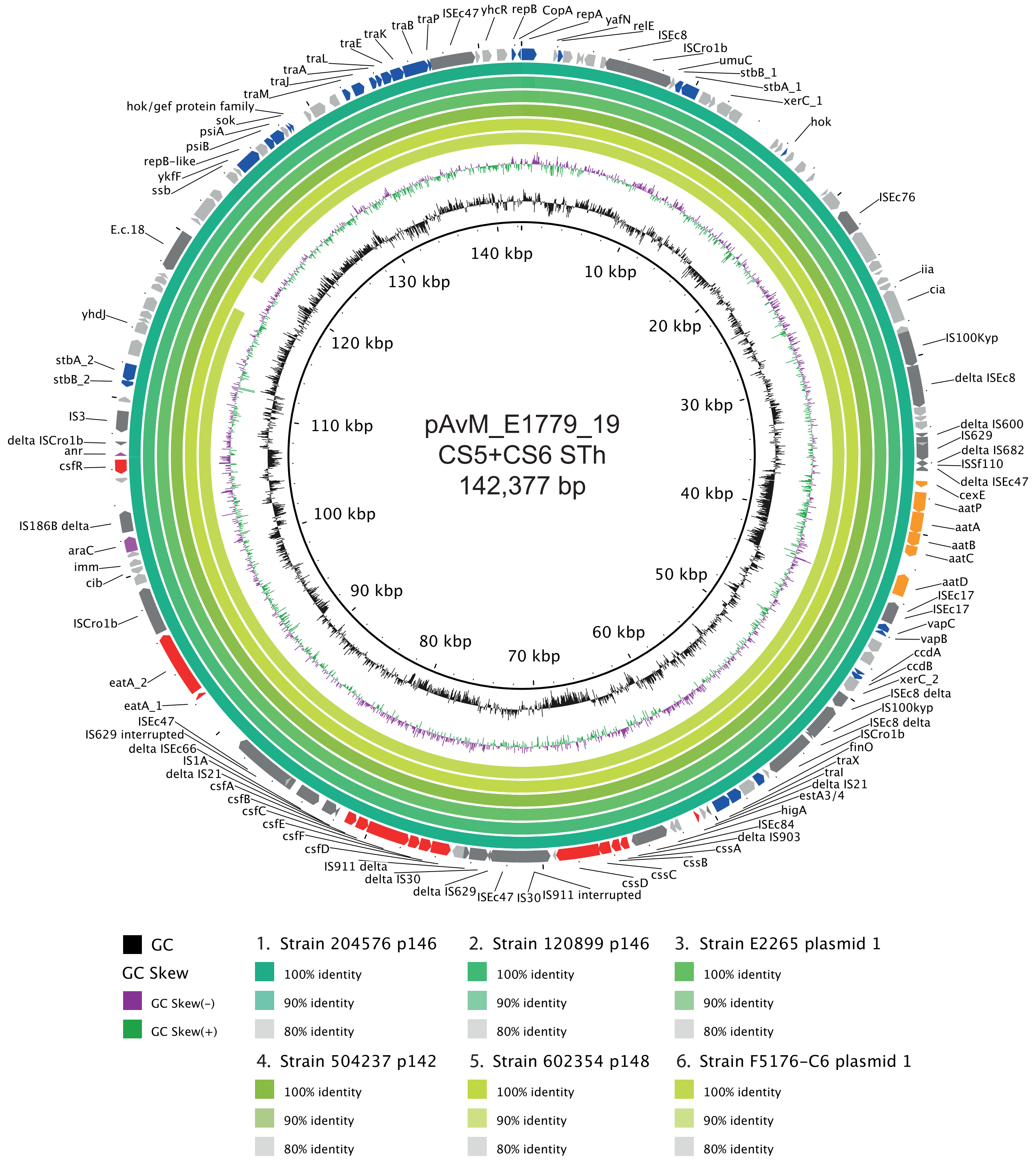

Figure S9: Comparison between the ETEC reference plasmids harbouring colonisation factors and other PacBio-sequenced ETEC plasmids using blastn. pAvM_E1779_19 (CS5+CS6) compared to 204576 p146 (2010, Mali; CP025908.1), 120899 p146 (2012, Gambia; CP025917.1), E2265 plasmid 1 (2006, Bangladesh; CP023347.1), 504237 p142 (2010, India; CP025863.1), 602354 p148 (2009, Bangladesh; CP025848.1) and F5176-C6 plasmid 1 (1997; CP024668.1). The figure was generated using BRIG (v0.95, URL: http://brig.-sourceforge.net/).

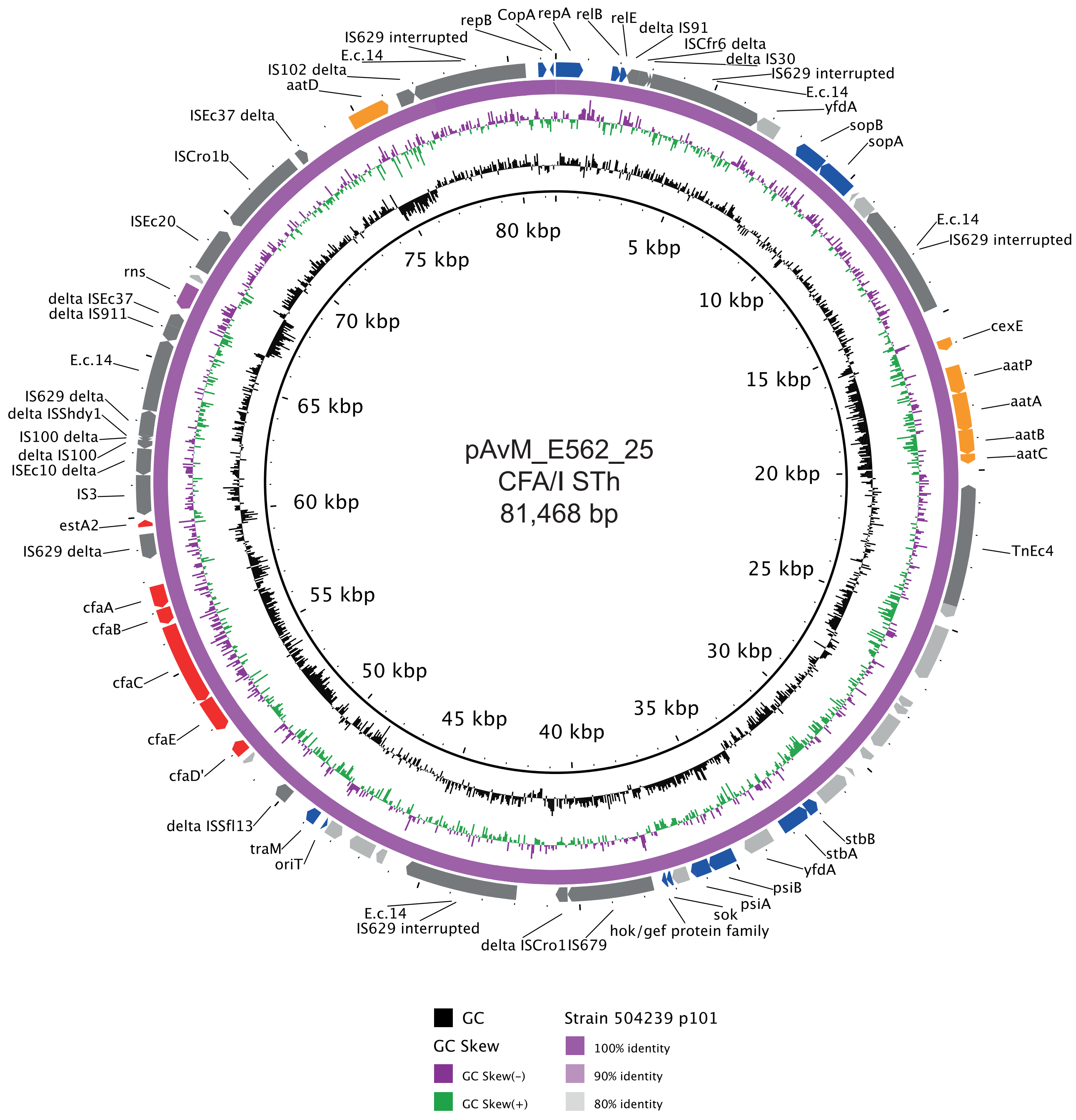

Figure S10: Comparison between the ETEC reference plasmids harbouring colonisation factors and other PacBio-sequenced ETEC plasmids using blastn. pAvM_E562_25 (CFA/I) compared to p504239_101 (2010, India; CP025860.1). The figure was generated using BRIG (v0.95, URL: http://brig.sourceforge.net/).

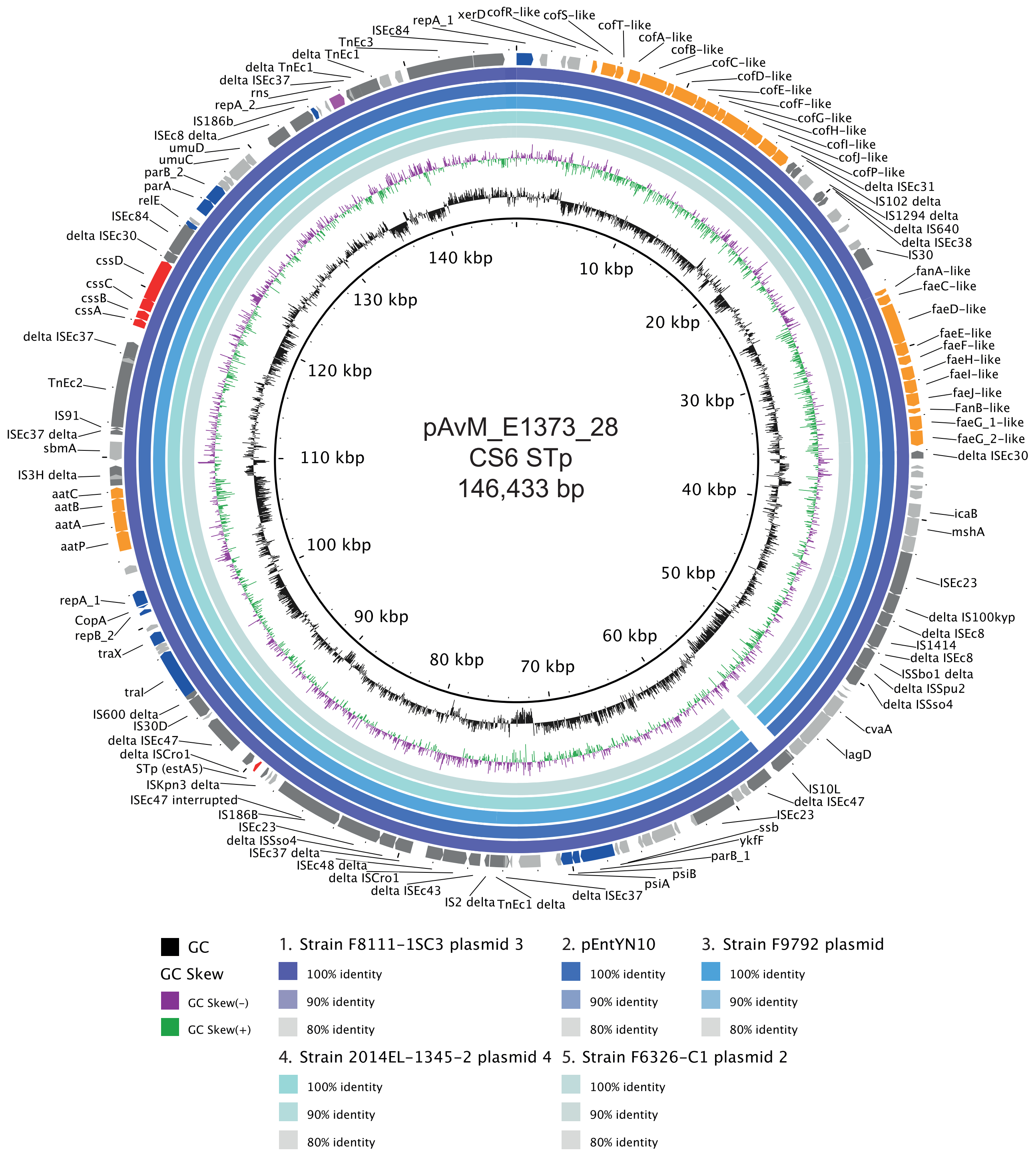

Figure S11: Comparison between the ETEC reference plasmids harbouring colonisation factors and other PacBio-sequenced ETEC plasmids using blastn. pAvM_E1373_28 (CS6) compared to F8111-1SC3 plasmid 3 (USA; CP024272.1), pEntYN10 (1991, Japan; AP014654.2), F9792 plasmid (USA; CP023274.1), 2014EL-1345-2 plasmid 4 (2014, USA; CP024227.1) and F6326-C1 plasmid 2 (1998, USA; CP024265.1). The figure was generated using BRIG (v0.95, URL: http://brig.sourceforge.net/).

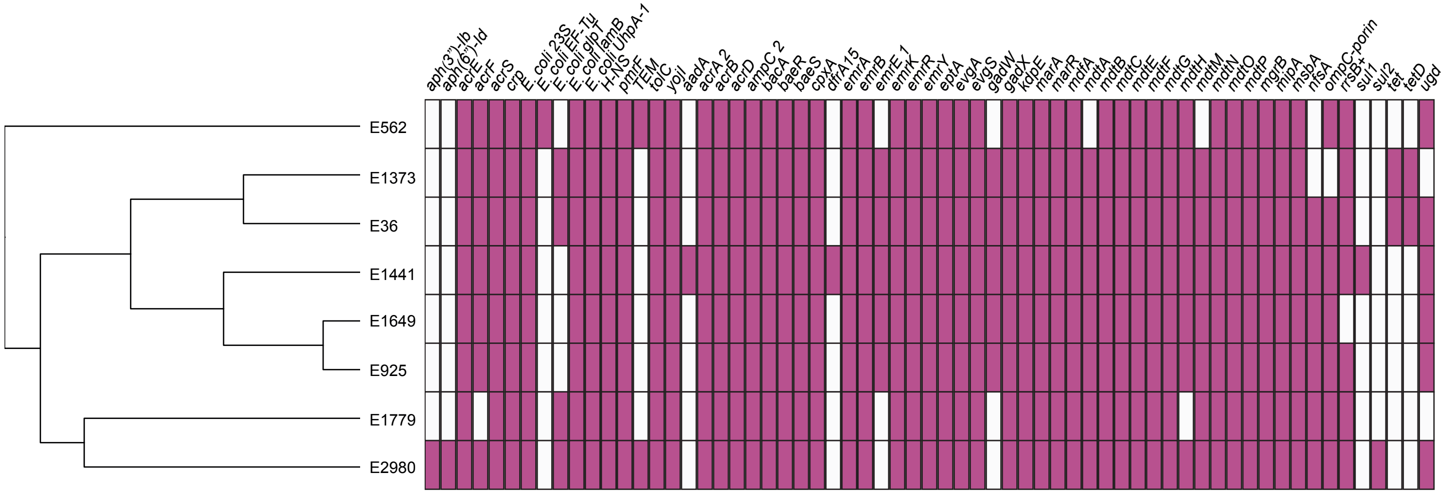

Figure S12: Presence and absence of antibiotic resistance genes. The figure was generated using Phandango (v1.3.0, URL: https://jameshadfield.github.io/phandango/#/).

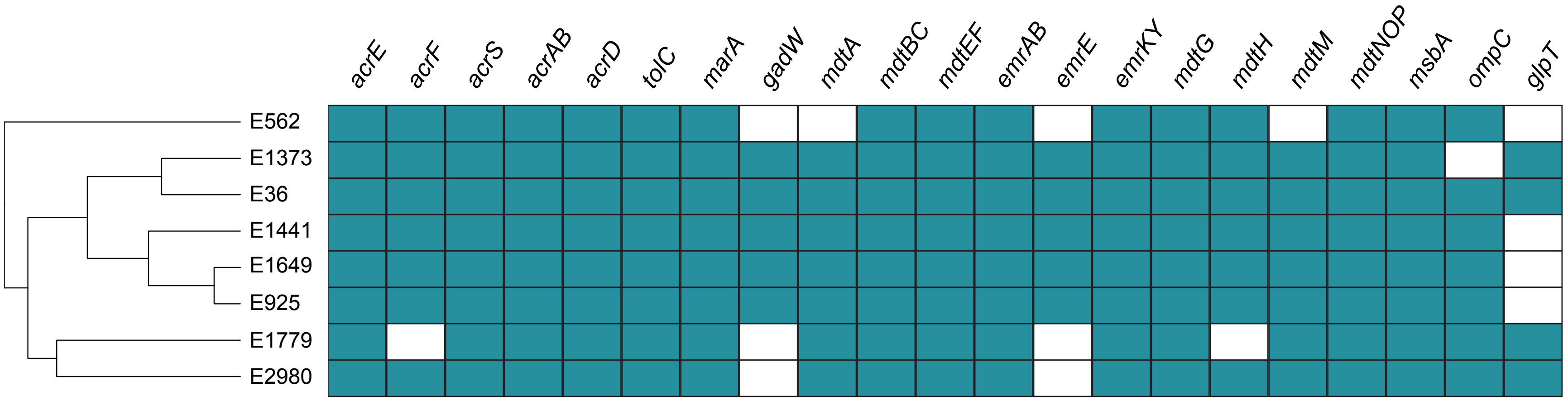

Figure S13: Presence and absence of efflux systems and porins. The figure was generated using Phandango (v1.3.0, URL: https://jameshadfield.github.io/phandango/#/).

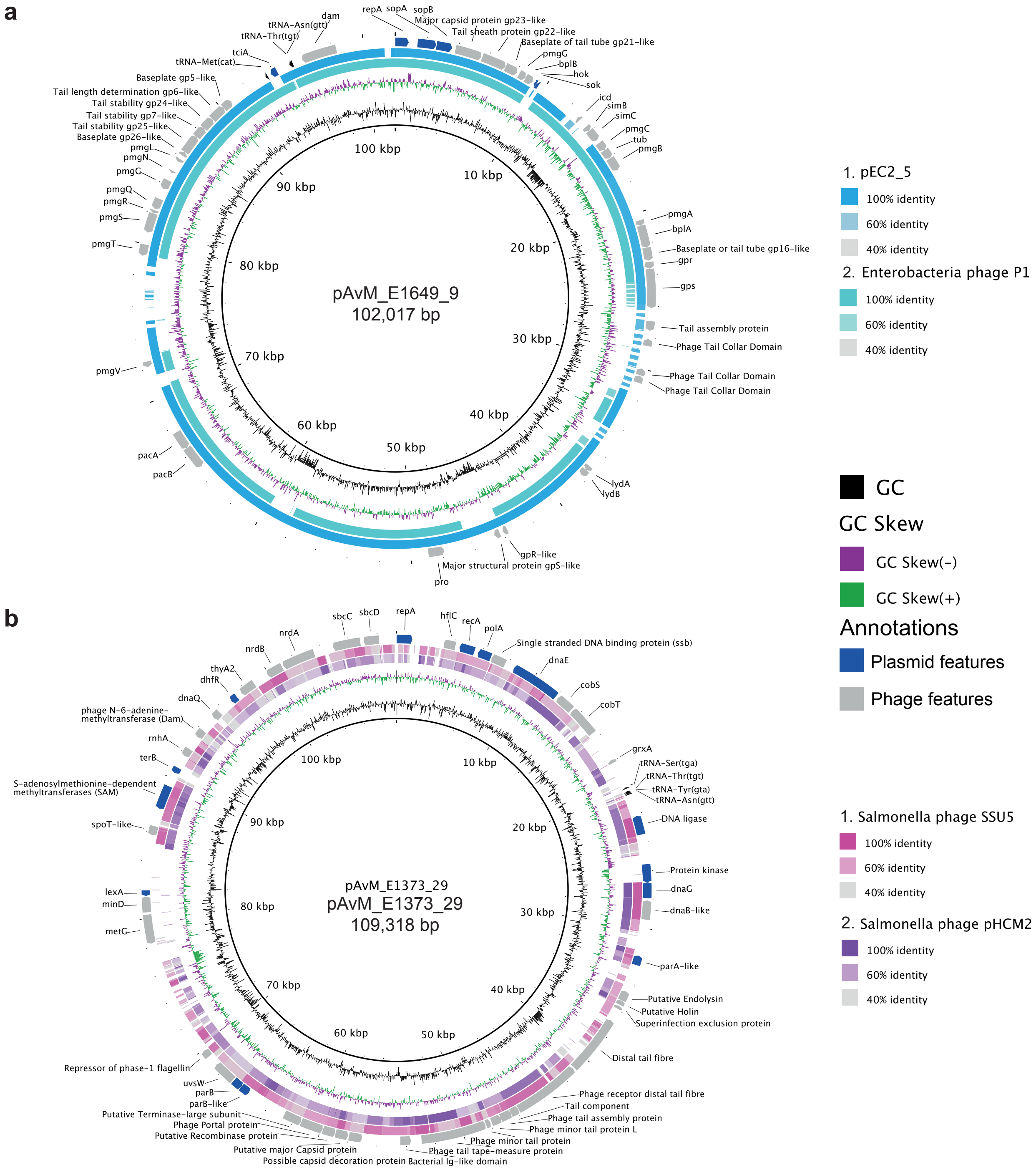

Figure S14: Comparison between the two identified ETEC phage-plasmids using tblastx. **a)** pAvM_E1649_9 is a P1-like phage plasmid here compared to pEC2_5 (*E. coli* strain EC2_5; CP041960.1) and Enterobacteria phage P1 (*Escherichia* virus P1; NC_005856.1). **b)** pAvM_E1373_29, a phage-plasmid similar to the SSU5 (Salmonella phage; JQ965645.1) and pHCM2 (*Salmonella typhi* strain CT18; AL513384.1) in *Salmonella typhi*. Tblastx comparison were made using BRIGS with the thresholds indicated to the right of each plasmid comparison. Annotations are from the respective ETEC phage-plasmids which were used as references for the blast. The figures were generated using BRIG (v0.95, URL: http://brig.sourceforge.net/).

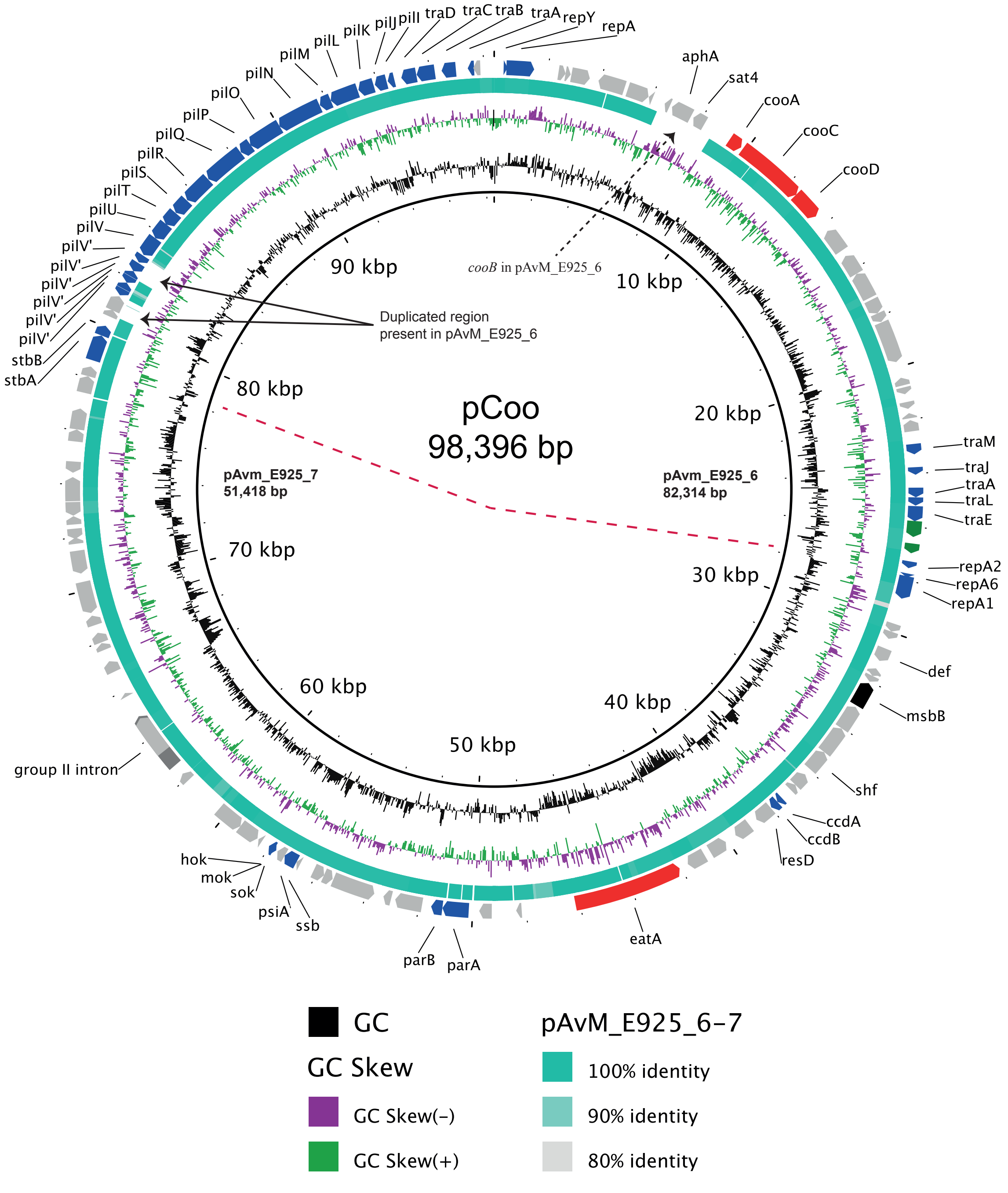

Figure S15: BLASTn comparison between the mosaic cointegrate pCoo (AY536429.1) and the concatenated plasmids pAvM_E925_6 and 7. The red dashed line indicates which part of the pCoo matches with which ETEC reference plasmid. An insertion and duplication have been introduced in pAvM_E925_6 as indicated by the two arrows. The gene *cooB* is missing from pCoo but is present in pAvM_E925_6, indicated with a dashed arrow. The image was generated using BRIG (v0.95, URL: http://brig.sourceforge.net/).

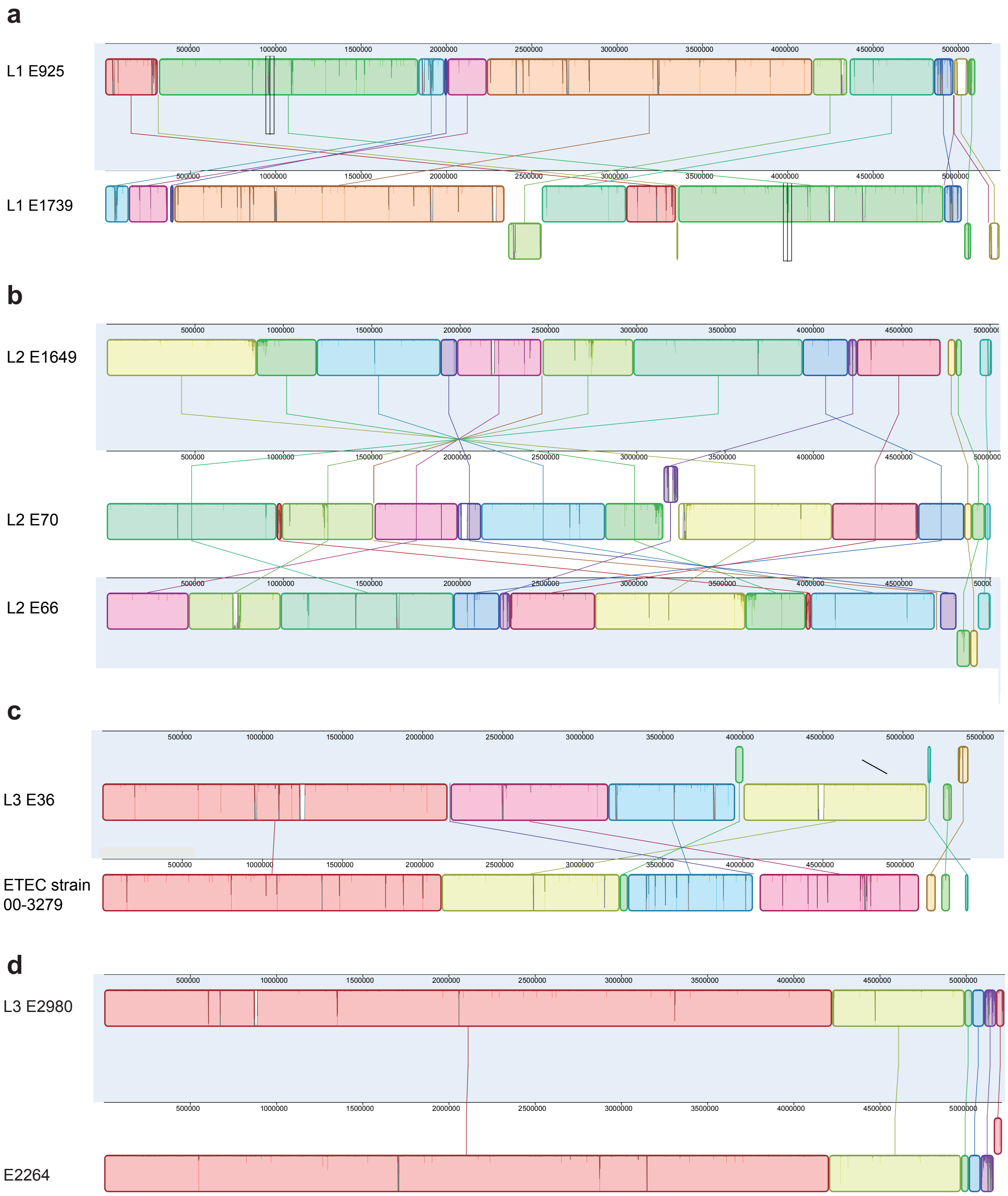

a
L1 E925
L1 E1739
b
L2 E1649
L2 E70
L2 E66
c
L3 E36
ETEC strain 00-3279
d
L3 E2980
E2264

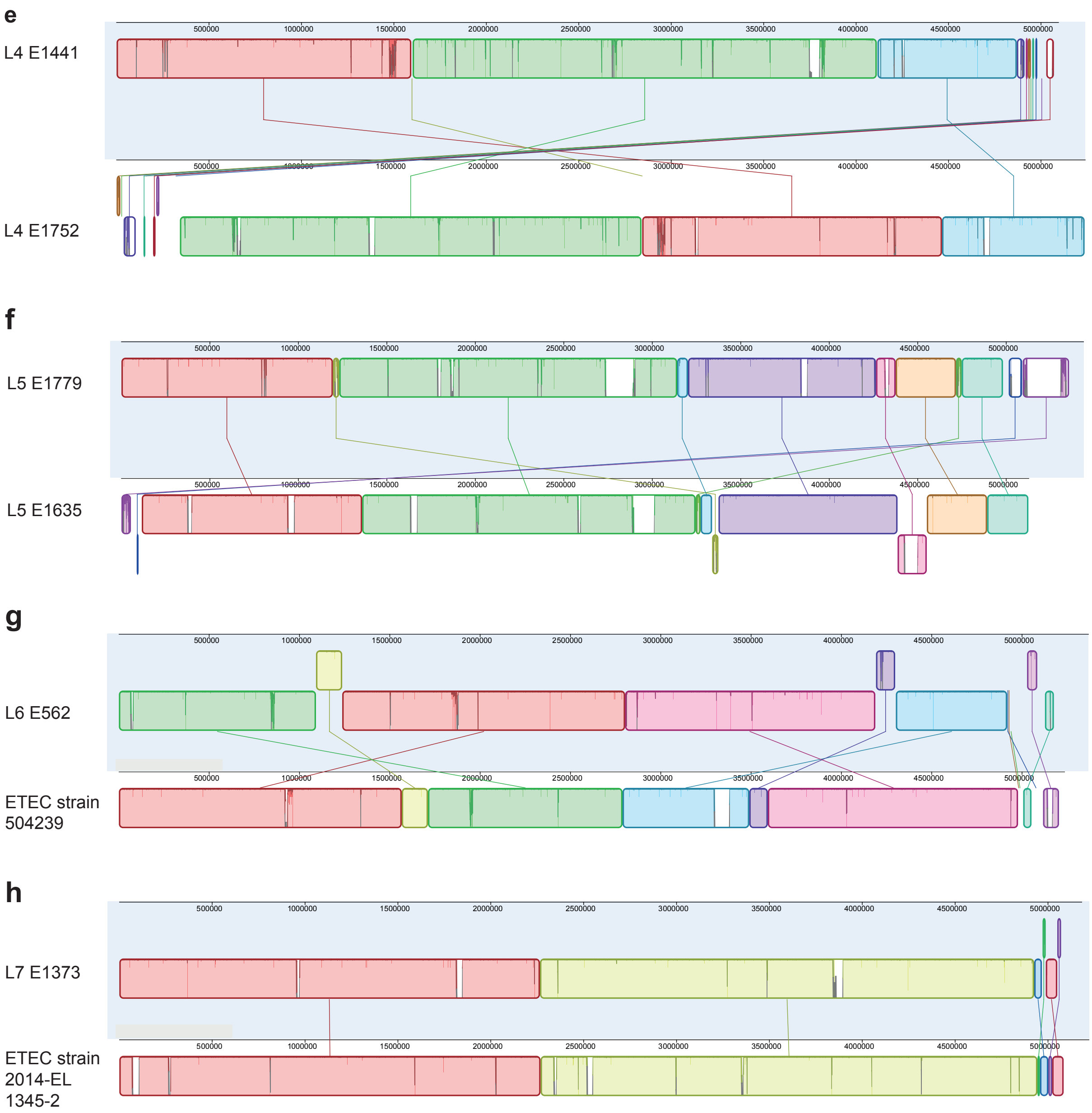

Figure S16: Whole genome comparisons using progressiveMAUVE between each ETEC reference strain with one or two different ETEC strains that belong to the same lineage. **a)** ETEC reference strain E925 compared to another long-read sequenced ETEC strain (E1739) that belong to the same lineage (L1). **b)** ETEC reference strain E1649, lineage 2, compared to two additional L2 ETEC strains (E70 and E66). **c)** ETEC reference strain E36, lineage 3, compared to ETEC strain 00-3279 (CP024293-96). **d)** ETEC reference strain E2980, lineage 3, compared to E2264 (CP023349-52). **e)** ETEC reference strain E1441 compared to E1752, both part of lineage L4. **f)** ETEC reference strain E1779 compared to E1635 part of lineage 5. **g)** ETEC reference strain E562 from lineage 6 compared to the ETEC strain 504239 (CP025859-61). **h)** ETEC reference strain E1373 part of lineage 7 compared to the ETEC strain 2014-EL-1345-2 (CP024223-27). The figures were generated using progressiveMauve (v2.4.0, URL: http://darlinglab.org/mauve/mauve.html).

Table S1: General characteristics of the chromosome of the ETEC reference strains

**Strain**	**L1 E925**	**L2 E1649**	**L3 E36**	**L3 E2980**	**L4 E1441**	**L5 E1779***	**L6 E562**	**L7 E1373**
**Length (bp)**	4,858,376	4,721,269	5,151,162	4,992,286	4,869,798	4,982,341	4,918,084	4,926,854
**GC (%)**	50.7	50.8	50.7	50.7	50.8	50.8	50.9	50.4
**Total no of CDSs**	4,521	4,409	4,816	4,924	4,560	4,649	4,568	4,760
**Accession number**	LR883050	LR882973	LR882997	LR882978	LR883012	LR883006	LR883000	LR882990

* Chromosome not circularized

Table S2: Phenotypic antibiotic profile of the ETEC reference strains.

**Strain**	**Lineage**	**MIC mg/L (R/I/S)***													
	Class	Penicillin			Cephalosporin		Tetracyclines		Fluoroquinolones		Macrolides		Sulphonamide	Misc agents	
	Antibiotic	Amp	Amc*	Oxa	Caz	Cro	Dox*	Tet*	Na*	Nor	Azm*	Ery*	Sxt	Cm	NIT
**E925**	L1	4 (S)	4 (S)	>128	0.5 (S)	0.5 (S)	2(S)	8 (I)	32 (R)	0.5 (S)	16 (S)	16	< 0.25 (S)	8 (S)	32 (S)
**E1649**	L2	8 (S)	8 (S)	>128	0.5 (S)	2 (I)	2 (S)	64 (R)	16 (S)	2 (S)	16 (S)	64	0.25 (S)	16 (I)	32 (S)
**E36**	L3	16 (I)	16 (I)	>128	0.5 (S)	2 (I)	64 (R)	2 (S)	16 (S)	1 (S)	16 (S)	64	S)	8 (S)	16 (S)
**E2980**	L3	>128 (R)	16(I)	>128	0.5 (S)	2 (I)	2 (S)	16 (R)	< 0.25 (S)	2 (S)	64 (R)	128	0.25 (S)	16 (I)	32 (S)
**E1441**	L4	4 (S)	4 (S)	>128	0.5 (S)	2 (I)	8 (I)	128 (R)	8 (S)	1 (S)	16 (S)	64	>128 (S)	>128 (R)	16 (S)
**E1779**	L5	16 (I)	16 (I)	>128	0.5 (S)	2 (I)	2 (S)	2 (S)	16 (S)	2 (S)	16 (S)	16	< 0.25 (S)	16 (I)	16 (S)
**E562**	L6	>128 (R)	16(I)	>128	0.25 (S)	1 (S)	8 (I)	128 (R)	8 (S)	0.5 (S)	16 (S)	32	< 0.25 (S)	8 (S)	32 (S)
**E1373**	L7	8 (S)	8 (I)	>128	0.5 (S)	2 (I)	64 (R)	>128 (R)	8 (S)	2 (S)	16 (S)	64	< 0.25 (S)	16 (I)	32 (S)

Amp = Ampicillin, Amc = Amoxicillin-clavulanic acid, Oxa = Oxacillin, Caz = Ceftazidime, Cro = Ceftriaxone, Dox = Doxucycline, Tet = Tetracyclin, Na = Naldixic acid, Nor = Norfloxacin, Azm = Azithromycin, Ery = Erythromycin, Cm = Chloramphenicol, NIT = Nitrofurantoin Sxt = sulphamethoxazole-trimethoprim

R = resistant; I = Intermediate; S = sensitive

*Clinical MIC breakpoints were not determined by EUCAST (The European Committee on Antimicrobial Susceptibility Testing. Breakpoint tables for interpretation of MICs and zone diameters. 2016., Version 9. http://www.eucast.org. [Accessed Febrary 2017] and The Clinical & Laboratory Standards Institute (CLSI).

Table S3: Genomic antibiotic resistance profile, including plasmid and chromosomal genes as well as efflux systems and porins and metal resistance

**Strain**	**Lineage**	**Antibiotic resistance genes**	**Porins and efflux pumps associated with antibiotic resistance***	**Metal resistance**
**E925**	L1	*ampC*, ampDE**	*acrRAB-tolC, acrEF-S, crp, emrRAB, emrE, mdf(A), gadW, mdtAB, mdtEF, mdtG, mdtH, mdtM, mdtNOP, msbA, ompC*	
**E1649**	L2	*ampC*, ampDE**	*acrRAB-tolC, acrEF-S, crp, emrRAB, emrE, gadW, mdf(A), mdtAB, mdtEF, mdtG, mdtH, mdtM, mdtNOP, msbA, ompC*	
**E36**	L3	*ampC*, ampDE*, tet(B)*	*acrRAB-tolC, acrEF-S, crp, emrRAB, emrE, gadW, glpT, mdf(A)-like, mdtAB, mdtEF, mdtG, mdtH,mdtM, mdtNOP, msbA, ompC*	
**E2980**	L3	*ampC*, ampDE*, blaTEM-1b, strA (aph(3'')-Ib-like), strB (aph(6)-Id), sul2*	*acrRAB-tolC, acrEF-S, crp, emrRAB, glpT, mdf(A), mdtAB, mdtEF , mdtG, mdtH, mdtM, mdtNOP, msbA, ompC*	
**E1441**	L4	*aadA1-like, ampC*, ampDE*, dfrA15, sul1 (Class I integron), tet(A) (Tn1721)*	*acrRAB-tolC, acrEF-S, crp, emrRAB, emrE, gadW, mdf(A)-like, mdtAB, mdtEF, mdtG, mdtH, mdtM, mdtNOP, msbA, ompC*	*mer* operon (*merRTPCADE*) (Tn21), *qacE* (part of the Class I integron)
**E1779**	L5	*ampC*, ampDE**	*acrRAB-tolC, crp, emrRAB, glpT, mdf(A)-like, mdtAB, mdtEF, mdtG, mdtM, mdtNOP, msbA*	
**E562**	L6	*ampC*, ampDE*, blaTEM-1b, tet(A)*	*acrRAB-tolC, acrEF-S, crp, emrRAB, mdf(A)-like, mdtEF, mdtG, mdtH, mdtNOP, msbA, ompC*	*mer* operon (*merRTPCADE*) (Tn21)
**E1373**	L7	*ampC*, ampDE*, tet(B)*	*acrRAB-tolC, acrEF-S, crp, emrRAB, emrE, glpT, mdf(A)-like, mdtAB, mdtEF, mdtG, mdtH, mdtM, mdtNOP, msbA*	

* Located on the chromosome

Table S4: Identified prophages in the chromosome and plasmids in each of the reference ETEC strains. Region of prophages and potential cargo genes are indicated.

Lineage/Strain/ CF profile	Chromosome/ plasmid	Prophage (Pph)	Source	Location[a]	Size (kB)	GC%	No. of proteins	Cargo genes
**L1 E925 CS1+CS3+CS21**	Chromosome	E925_Pph_1	Entero_P88_ NC_026014(25)	1980800-2010357	29.6	50.1	39	flagella biosynthesis regulator (ORF: 01987)
		E925_Pph_2 (Cryptic)	Entero_lambda_ NC_001416(6)	2829055-2849450	23.4	50.4	24	*ompX* and *sohB* (probable Protease)
		E925_Pph_3 (cryptic)	Entero_lambda_ NC_001416(4)	3229100-3240718	11.62	44.1	15	Outer membrane lipoprotein, *blc*
		E925_Pph_4	Shigel_SfIV_ NC_022749(12)	3724871-3749140	24.3	46.7	32	*gadW* transcriptional regulator of the AraC family (helix-turn-helix motif). tRNA-Thr insertion site
	pAvM_E925_7	E925_Pph_5	Stx2_c_1717_ NC_011357(3)	23316-45489	22.1	48.7	22	*eatA_3, eatA_4, eatA_5* (*eatA* disrupted in three ORFs), *stbAB* and *psiA*
**L2 E1649 CS2+CS3+CS21**	Chromosome	E1649_Pph_1 Cryptic phage	Escher_TL_2011 b NC_019445(29)	1298543-1335201	36.6	49.1	45	*istB*-like ATP binding protein similar to DNA replication protein, *dnaA*.
		E1649_Pph_2	Entero_P88_ NC_026014(33)	1502430-1542242	29.1	52.4	46	Putative Acetyltransferase and arabinose transporter
		E1649_Pph_3 (cryptic phage)	Entero_P88_ NC_026014(15)	2177200-2195400	17.7	49.9	23	No cargo genes
		E1649_Pph_4	Entero_P4_ NC_001609(9)	4048900-4077099	21.05	48.4	26	*nanC*- Porin gene (N-acetylneuraminate epimerase precursor and putative inner membrane protein). tRNA-Leu insertion site

	pAvM_E1649_ 9	E1649_Pph_6	Salmon_SJ46_ NC_031129(88)	4916371-5038771	122.4	47	138	*eatA*. This is the P1 like phage-plasmid
**L3 E36 CFA/I + CS21**	Chromosome	E36_Pph_1 (cryptic phage)	Entero_P4_ NC_001609(9)	47658-59450	11.6	48	15	Retron-type Reverse transcriptase and DNA primase
		E36_Pph_2	Entero_P4_ NC_001609(9) and Salmon_Fels_2 NC_010463(37)	Two adjacent phages: 1220220-1231640 and 1231677-1266590	46.4	49.6	61	Putative bacteriocin adjacent to the *intA_2* gene (ORF: 01826). Insertion site is in the region of *ssrA*
		E36_Pph_3	Entero_P88_ NC_026014(41) P2 family of bacteriophage	1285100-1328400	43.3	52.7	56	stbA and stbB (plasmid stability genes) and *tfp* pilus assembly gene*, pilA*
		E36_Pph_4	Entero_mEp460_ NC_019716(23	2138535-2199525	48.0	50.3	63	*terB* (tellurite reistance), and *ompX.* Insert site is tRNA-Gly
		E36_Pph_5	Entero_mEp460_ NC_019716(23) (probable cryptic phage)	2499123-2535609	36.5	50.9	40	No cargo genes
		E36_Pph_6	Burkho_BcepMu _NC_005882(32)	2738731-2795496	56.7	52.4	68	Potassium transporter, *trkG* and *parB* partition protein (with nuclease N-terminal domain)
		E36_Pph_7	Entero_HK629_ NC_019711(21)	3024129-3076300	36.5	51.3	56	Tellurite resistance protein and ncRNA, *dicF* and *dicC* genes present are of interest but may not be considered as cargo).
		E36_Pph_8	Entero_lambda_ NC_001416(27)	3532592-3576100	43.5	51.6	71	Outer membrane protein Blc. Group II intron reverse transcriptase (*ltrA*_14) with adjacent group II intron *uvrB*, *sohB_3, dicC* (ORF: 04216)

		E36_Pph_9	Entero_P4_ NC_001609(12)	44922786- 4519904	27.6	49.8	14	No cargo genes
	pAvM_E36_12	E36_Pph_10 (cryptic phage)	Entero_BP_4795_ NC_004813(3)	182814- 195964	13.1	52.9	23	No cargo genes
		E36_Pph_11 (cryptic phage)	Entero_BP_4795_ NC_004813(3)	267737- 281095	13.3	51.2	21	*sopB* and *estA2*
	Prophage* Contig 2	E36_Pph_12 (Partial - no *int* gene present. Probable cryptic)	Salmon_Fels_2_ NC_010463(24)	700-18544	17.8	55.4	29	No cargo genes
	Prophage* Contig 998	E36_Pph_13 Cryptic phage)	Entero_lambda_ NC_001416(11	1-16565	16.5	54.2	22	No cargo genes
**L3 E2980 CS7**	Chromosome	E2980_Pph_1	Salmon_SEN34_ NC_028699(24)	1314690- 1354910	40.22	49.7	50	No cargo genes
	Chromosome	E2980_Pph_2	Shigel_Sf6_ NC_005344(14)	1568700- 1611651	42.9	46.4	63	*wzy* (ORF: 01970) gene is an oligosaccharide repeat unit polymerase adjacent to the integrase (*oac* gene in sf6 phage at the same location next to *int*).tRNA-Arg insertion site
	Chromosome	E2980_Pph_3	Entero_P88_ NC_026014(43)	2070150- 2109664	39.5	51.6	55	Putative plasmid stability proteins and *repA* (ORFs: 02444, 02445 and 02446) and flagella biosynthesis regulator (as found in the P88 phage of E925 above) near *int* gene. tRNA-Leu insertion. This pahge is related to P1 phage so it can probably exist as a Phage-Plasmid.
	Chromosome	E2980_Pph_4 (Cryptic phage)	Entero_lambda_ NC_001416(11)	2447775- 2466400	18.6	51.8	17	No cargo genes

	Chromosome	E2980_Pph_5	Entero_lambda_ NC_001416(22)	2918700-2962650	43.95	51.6	60	*tciAB* (tellurite resistance), *ompX*
	Chromosome	E2980_Pph_6	Entero_lambda_ NC_001416(27)	3588788-3636557	47.8	49.6	58	No cargo genes. tRNA insertion site adjacent to *int* gene, *ompX* gene
	Prophage* Contig 5	E2980_Pph_7	Salmon_SEN34_ NC_028699(13)	8730-21920	13.1	49.4	25	No cargo genes
**L4 E1441 CS6**	Chromosome	E1441_Pph_1	Salmon_HK620_ NC_002730(17)	1473781-1517255	43.5	46.5	57	tRNA-Arg insertion site. CatB-related O-acetyltransferase adjacent to *int*
	Chromosome	E1441_Pph_2	Entero_mEp460_ NC_019716(11)	2663330-2710354	47.0	50.6	65	Anti-adapter gene *iraM* (operates via *rpoS*)
	Chromosome	E1441_Pph_3	Entero_lambda_ NC_001416(26)	3194858-3250061	55.2	50.4	66	Phospholipid binding protein, *ybhB*; Inner membrane protein, yeaI; *slyB* lipoprotein precursor, *sohB;* outer membrane protein, *ompD and slyB* and *kilR*
	Chromosome	E1441_Pph_4	Entero_mEp460_ NC_019716(37)	3756217-3834601	78.38	52	101	CysO. Phage adjacent to tRNA-Thr
**L5 E1779 CS5+CS6**	Chromosome	E1779_Pph_1	Salmon_Fels_2_ NC_010463(36)	1195952-1232915	36.9	49.1	45	Cargo genes are present but all the hypothetical. *ssrA* integration site.
	Chromosome	E1779_Pph_2	Entero_P2_ NC_001895(21)	1783098-1807397	24.3	49.8	32	No cargo genes identified
	Chromosome	E1779_Pph_3	Entero_lambda_ NC_001416(22)	2368430-2397000	28.57	55.4	37	*sohB*-probable protease
	Chromosome	E1779_Pph_4	Entero_lambda_ NC_001416(18)	3157352-3201305	43.9	51.6	56	*iraM* (anti-adaptor protein blocking RpoS degradation)
	Chromosome	E1779_Pph_5	Entero_cdtI_NC_ 009514(22)	2859100-2906257	47.16	51.6	65	Some potential cargo genes but all hypothetical

	Chromosome	E1779_Pph_6	Entero_lambda NC_001416(29)	3288450-3335837	47.4	51.5	65	Putative *ompD* porin protein, ORF: 03897 encodes ZapA, a cell division protein
	Chromosome	E1779_Pph_7	Shigel_SfII NC_021857(35)	3839659-3878224	38.5	50.8	55	tRNA-Thr insertion site. Region with number of regulators: HTH-type transcriptional regulator *gadW*-like (acid resistance).lexA repressor*, perC*-family activator*, gntR* family regulators, *yfdR* family of *dicC* DNA binding transciprtional regulator, lexA/HTH-type transcriptional regulator; *yfbR*-5' deoxynucleotidase
	Chromosome	E1779_Pph_8	Entero_fiAA91_ss_NC_022750 (31)	4718700-4750300	31.6	51.7	42	No cargo genes
	pAvM_E1779_19	Phage islet	Stx2_c_1717_ NC_011357(3)	49007-62586	13.5	51.3	23	*estA3/4*, *traI*, *traX*, *finO*
	Probable phage-plasmid	E1779_Pph_9	Entero_N15_ NC_001901(21)	1-46243	46.2	50.5	69	*ompX* (resistance to complement killing), Phage-specific genes
**L6 E562 CFA/I+CS21**	Chromosome	E562_Pph_1	Entero_lambda_ NC_001416(22)	2695572-2745700	49.4	51.5	62	*ftsZ* inhibitor gene, *kilR.* Though may be of phage origin.
	Chromosome	E562_Pph_2	Entero_fiAA91_ss NC_022750(31)	4666803-4698527	31.7	52	42	No cargo genes
**L7 E1373 CS6**	Chromosome	E1373_Pph_1	Entero_mEp460 NC_019716(23)	2848300-2890275	41.98	51.5	22	*ompX* (resistance to complement killing), Tellurite resistance gene, *tehB and a S*-Adenosyl-l-methionine (SAM)-dependent
	Chromosome	E1373_Pph_2	Shigel_SfIV NC_022749(34)	3856500-3897232	40.73	48.7	59	tRNA-Thr insertion site. *kilA*-like domain, *lexA, perC* and

			Similar to the content of E1779_Pph_7 (Shigel_SflI_NC_021857(35))					*gntR* regulator. An almost identical region is present in the *Shigella* phage related to SflV (accession number KC814930.1)
	pAvM_E1373_29	E1373_Pph_3	Salmon_SSU5_NC_018843(91) This is the Phage-Plasmid related to pHCM2 and SSU5	1-109318	109.1	46.4	130	*terB*, mind (cell division inhibitor), *uvrB*, *parB*-like (2 genes, ORFs:04807 and 04808), *hlyB*, Flp pilus assembly (ORF: 04842), *grxA* (glutaredoxin), *cobT* and *cobS* (aerobic cobaltochelatase subunits), *dnaE, polA* (DNA polymerase I), recA (recombiase A), *repA* (FIB), *nrdA* and *nrdB* (ribonucleoside-diphoasphatase), a lot of hypo proteins.

[a]The location within the chromosome, plasmid or contig individually.
