# Supplementary material for: Long-read-sequenced reference genomes of the seven major lineages of enterotoxigenic Escherichia coli (ETEC) circulating in modern time
Source: Sci Rep. 2021 Apr 29;11:9256. doi: 10.1038/s41598-021-88316-2 (PMC8085198; doi:10.1038/s41598-021-88316-2)
Supplement: Supplementary file 6 — Supplementary Information 6. [file 41598_2021_88316_MOESM6_ESM.docx]

**Additional information**

**Additional file 1:** Supplemental Figures and Tables

**Additional file 2:** Detailed description of ETEC reference plasmids

**Additional file 3:** Excel file with plasmid classification – Inc groups

**Additional file 4:** Excel file with metadata of ETEC and *E. coli* genomes included in the phylogenetic tree

**Additional file 5:** Excel file with a compiled list of all accession numbers for chromosomes and plasmids
